# Anti-Inflammatory Potency of Human Wharton’s Jelly Mesenchymal Stem Cell-Derived Exosomes on L2 Cell Line Induced by Lipopolysaccharides

**DOI:** 10.34172/apb.2024.027

**Published:** 2024-01-13

**Authors:** Ika Adhani Sholihah, Anggraini Barlian

**Affiliations:** ^1^School of Life Sciences and Technology, Institut Teknologi Bandung, Jl. Ganesha No.10, Bandung 40132, Indonesia.; ^2^Research Center for Nanosciences and Nanotechnology, Institut Teknologi Bandung, Bandung, West Java 40132, Indonesia.

**Keywords:** Anti-inflammatory, Exosomes, Hypoxia, Interleukin-1β, hWJ-MSCs, Tumor necrosis factor α

## Abstract

**Purpose:**

At present, therapeutic interventions to treat acute lung injury (ALI) remain largely limited to lung-protective strategies, as no real molecular-driven therapeutic intervention has yet become available. The administration of bacterial lipopolysaccharides (LPS) is known as an inflammatory activator, representing a frequently used model of ALI. This study investigated the biological function of normoxic (21% O_2_ ) vs. hypoxic conditions (5% O_2_ ) obtained from human Wharton’s Jelly mesenchymal stem cells (hWJ-MSCs) and discovered that exosomes have the ability to suppress inflammatory responses by specifically targeting TNF-α, IL-1β, IL-6. and identify the toll-like receptor 4 (TLR4) NF-κβ gene expression.

**Methods:**

Primer culture hWJ-MSCs characterization with trilineage differentiation and CD markers was conducted. To obtain exosomes, hWJ-MSCs were stimulated with two different oxygen levels: 21% (nor-exo) and 5% (hypo-exo). Then, the L2 cell line was induced with LPS 1 µg/mL. Inflamed-L2 was treated with nor-exo, hypo-exo, and dexamethasone as a positive control. The RNA extracted from treated L2 cells was utilized to examine the gene expression profiles of TLR4 and NF-κβ, and the medium was used to measure tumor necrosis factor α (TNF-α), interleukin (IL)-1β, and IL-6 levels using ELISA. Lastly, proteomic analysis of the exosome using LC/MS-MS was conducted.

**Results:**

Nor-exo and hypo-exo can be characterized and can produce higher yields exosomes under hypoxic conditions. The expression of TLR4 and NF-κβ genes and the proinflammatory levels such as IL-6, IL-1β, and TNF-α levels in nor-exo and hypo-exo treatments decreased.

**Conclusion:**

Nor-exo and hypo-exo derived from hWJ-MSCs were proven to have anti-inflammatory activities.

## Introduction

 Acute lung injury (ALI) is characterized by the disruption of the alveolar–capillary barrier and the presence of significant prevalent inflammation.^1,2^ Although critical care has made significant progress, there is currently no drug that effectively targets the underlying pathophysiology of ALI.^3^ A gram-negative bacterial component known as lipopolysaccharide (LPS) has been identified as systemic infections in the ALI model. It binds to the toll-like receptor 4 (TLR4) on the surface of the cell membrane, activates the nuclear factor kappa-B (NF-κβ) signaling pathway and induces inflammatory cascades. LPS can also activate inflammatory signaling in the lung via the systemic circulation when the epithelial barrier is damaged and tissue permeability increases, thereby expanding immune responses.^4^ Damage to lung tissues is also linked to large numbers of immune cells moving into an inflamed lung. A significant amount of proinflammatory substances, including tumor necrosis factor α (TNF-α), interleukin-1β (IL-1β), interleukin-6 (IL-6), and proteases, is released by active neutrophils and alveolar macrophages, causing the damage to lung tissues more severe.^5^ Protecting the epithelial barrier and suppressing inflammation in the lungs may prevent the acute immune response that happens in lung diseases.^6^

 Mesenchymal stem cells-derived exosomes (MSCs-Exos) have demonstrated potential as a cell-free therapy for ALI.^7^ Previous research shows that MSCs-Exos decrease inflammation through NF-κβ regulation. Further investigations demonstrate that MSCs-Exos contain miR-182-5p and miR-23a-3p, targeting the inhibitor of nuclear factor kappa-B kinase subunit beta (Ikbkb) and ubiquitin-specific peptidase 5 (Usp5) genes and encoding IKKβ and Usp5 proteins, respectively. Their silencing causes a decrease in IKKβ expression and an increase in its ubiquitination, leading to the coinhibition of the NF-κB pathway.^8^ Liu et al showed that human umbilical mesenchymal stem cells-Exo (hUMSCs-Exo) reduced the TNF-α, IL-1β, and IL-6 levels in a burn-induced lung injury rat model. This effect was achieved by miR-451 by suppressing the toll-like receptor 4 (TLR4)/NF-κB signaling pathway.^9^ Similar to human umbilical cord mesenchymal stem cells (hUC-MSCs), human Wharton’s jelly mesenchymal stem cells (hWJ-MSCs) can be used as a source of exosomes. hWJ-MSCs have stem cell properties and are easy to extract from the umbilical cord.^10^ In addition, the number of hWJ-MSCs samples is high compared to other sources.^11^

 Nowadays, such small yield of exosomes has been an obstacle for the expansion of basic research regarding exosome analysis and applications in drug delivery.^12^ One way to increase the yield of exosomes is using a hypoxic treatment approach in hWJ-MSCs. Hypoxia induces an increase in the number of exosomes in cells. Cargo sorting, transport of MVBs, and fusion with the plasma membrane are the key steps in exosome release, and hypoxia may influence these steps. Cargoes and cargo-sorting machinery are the first regulators of exosome release, and hypoxia may mediate their activity.^13^ Therefore, our purpose is to determine the biological role of exosomes derived from hWJ-MSCs with different oxygen levels of 21% (normoxia) and 5% (hypoxia), to elucidate how these exosomes can potentially inhibit inflammatory responses, and to identify the TLR4 gene expression as the first barrier for LPS attachment in L2 cell line and NF-κβ for the inflammatory pathway. Proteomic analysis was performed to support the obtained data and to give a broader insight in this study.

## Materials and Methods

###  Materials 

 The exosome isolation kit (Invitrogen^TM^, 4478359, USA) and CD63 ELISA kit (Invitrogen^TM^, EH95RBX5, USA) were purchased from PT. Nutrilab Pratama. Rat TNF-α (Elabscience, E-EL-R2856, USA), rat IL-6 (Elabscience, E-EL-R0015, USA), and rat IL-1β (Elabscience, E-EL-R0012, USA) were purchased from PT. Inti Kemika Sejahtera. Dulbecco’s Modified Eagle’s Medium (DMEM) High Glucose medium (Sigma-Aldrich, D5796, Germany), Antibiotic Antimycotic (Sigma-Aldrich, A5955, Germany), and amphotericin B (Sigma-Aldrich, A2942, Germany) were purchased from PT. Elo Karsa Utama.

###  Isolation of human Wharton’s jelly mesenchymal stem cells ( hWJ -MSCs)

 Human umbilical cords were collected in RS Dr. Hasan Sadikin, Bandung, Indonesia, from newborns after obtaining parental permission. All experimental procedures and manipulations were in compliance and were approved by the Medical Research Ethics Committee of Universitas Padjajaran, Indonesia (approval letter no. 477/UN6.KEP/EC/2021; date of approval: June 09, 2021).

 As described in a previous study, hWJ-MSCs cells were isolated using the explant method.^14^ Umbilical cord of 10–15 cm length was washed with sterile PBS, and then, the arteries were removed from the Wharton’s jelly part. The umbilical cord tissue was cut into approximately 0.5–1 cm pieces and then attached to a 100 mm tissue culture plate. Once attached, 5 mL of growth medium was carefully added around the tissues. The growth medium was DMEM high glucose medium (Sigma-Aldrich, D5796, Germany) containing 10% fetal bovine serum (Gibco^TM^, 16000044, USA), 1% Antibiotic Antimycotic (Sigma-Aldrich, A5955, Germany), and 1% amphotericin B (Sigma-Aldrich, A2942, Germany). Culture cells were incubated at 37 °C with 5% CO_2_, and the medium was replaced every 2 days. After approximately 10–12 days, when cells with a fibroblast-like morphology migrated from the explant, the tissues were removed. Once the primary culture (P0) reached 80% confluency, the cells were expanded until passage 4 (P4).

###  Characterization of hWJ -MSCs

 The hWJ-MSCs primer culture requires characterization to confirm that the isolate consists of mesenchymal stem cells (MSCs). This can be achieved using two procedures: trilineage differentiation and cell surface marker analysis. MSCs must have CD73, CD90, and CD105 gene expressions and must have the potential to differentiate into adipocytes, chondrocytes, and osteocytes.^15^ The trilineage experiment used hWJ-MSCs passage 4 (3 × 10^4^ cells/well) on a 12-well plate. Once the confluency reached approximately 80%, adipogenic (StemPro^TM^, A1007001, USA), chondrogenic (StemPro^TM^, A1007101, USA), and osteogenic (StemPro^TM^, A1007201, USA) media were substituted for the growth medium. After 21 days at 37 °C and 5% CO_2_, cells were fixed with 4% formaldehyde for 30 min and the medium was washed three times with PBS 1 × (Sigma-Aldrich, D8537, Germany). After washed with PBS, staining was carried out, lipid droplet formation (adipogenic differentiation) stained with oil red o (0.3% in 2: 1 isopropanol: dH_2_O), glycosaminoglycan (GAG) accumulation (chondrogenic differentiation) stain with alcian blue (1% in 0.1 N HCl), and calcium deposits (osteogenic differentiation) stained with alizarin red (2% in dH_2_O, pH 4.2) for 30 minutes.^14^ Next, the cells were rinsed using distilled water three times and observed using an inverted microscope.

 Analysis of cell surface marker expression was carried out by resuspending the cells with 4.5 mL of FACS Buffer (9 reactions). Staining was performed using the Stemflow^TM^ hMSC Analysis Kit as follows: (1) cell only (500 µL), (2) single staining-FITC (5 µL), (3) single staining-PE CD44 (5 µL), (4) single staining-PerCP (5 µL), (5) single staining-APC (5 µL), (6) isotype negative (isotype positive 20 µL + Isotype negative 20 µL), (7) isotype positive (Isotype 20 µL and PE IgG 5 µL), (8) mix cocktail negative CD44 (positive cocktail 20 µL and negative cocktail 20 µL), (9) mix cocktail CD44 (positive cocktail 20 µL and PE CD44 5 µL). After that, characterization was carried out using a flow cytometer.

###  Isolation and identification of hWJ -MSC-Exo

 The hWJ-MSCs cells cultured with DMEM high glucose medium (Sigma-Aldrich, D5796, Germany) containing 10% fetal bovine serum (Gibco^TM^, 16000044, USA), 1% Antibiotic Antimycotic (Sigma-Aldrich, A5955, Germany), and 1% amphotericin B (Sigma-Aldrich, A2942, Germany). hWJ-MSCs cells that had reached passage 4 had their medium replaced with 5 mL DMEM low glucose medium (Sigma-Aldrich, D6046, Germany) without fetal bovine serum in a T25 flask and were then treated in an incubator with different oxygen levels, namely, 21% (normoxia) and 5% (hypoxia) for 24 hours at 37 °C. After 24 hours, conditioned medium was taken, which contained exosomes secreted by hWJ-MCs. A total exosome isolation reagent (Invitrogen^TM^, 4478359, USA) was used for isolating exosomes according to the manufacturer’s protocol. The expression of protein CD63 on nor-exo and hypo-exo was used human CD63 ELISA kit (Invitrogen^TM^, EH95RBX5, USA) according to the manufacturer’s protocol. Exosome concentration and particle size were analyzed using nanoparticle tracking analysis (NTA), which was performed on a NanoSight NS300 system (Malvern Instruments, Malvern, UK), and exosomes morphological characteristics were identified using transmission electron microscopy (TEM) HT7700 (Hitachi, Japan).

###  L2 cell line culture

 The rat type II alveolar epithelial cell line L2 (ATCC, CCL-149^TM^, USA) was cultured in DMEM High Glucose medium (Sigma-Aldrich, D5796, Germany) containing 10% fetal bovine serum (Gibco^TM^, 16000044, USA), 1% Antibiotic Antimycotic (Sigma-Aldrich, A5955, Germany), and 1% amphotericin B (Sigma-Aldrich, A2942, Germany). The medium was changed every 48 hours.

###  Exosome labeling and uptake in L2 cells

 Purified nor-exo and hypo-exo were labeled with 250 μL of Diluent C and 1 μL of PKH67 (Sigma-Aldrich, PKH67GL, Germany) for 4 min with moderate agitation. PKH67 was used to stain exosome membranes, and Diluent C was used to improve efficient labeling. Labeled nor-exo and hypo-exo were centrifuged at 13.000 g at 4 °C for 1 hour to remove excess dye. Final pellets were resuspended in DMEM high glucose, and after 6 and 24 hours in L2 cells, the dilabeled exosomes were fixed in 4% paraformaldehyde and stained with DAPI. The confocal laser scanning microscope Olympus FV-1200 was used to measure fluorescence distribution and intensity to examine the L2 cell uptake of exosomes.

###  Experimental

 On the basis of the particle data using NTA, the concentrations of nor-exo and hypo-exo were determined to be 3.125 (3.125 × 10^4^) for the lower concentration and 10 (1 × 10^6^ particle/mL) for the higher concentration. To assess the effect of nor-exo and hypo-exo, L2 cells were divided into seven subgroups: normal cell, negative control (LPS 1 µg/mL), positive control (LPS 1 µg/mL + Dex 40 ng/mL), nor-exo 3.125 (LPS 1 µg/mL + 3.125 × 10^4^ particle/mL), nor-exo 10 (LPS 1 µg/mL + 1 × 10^6^ particle/mL), hypo-exo 3.125 (LPS 1 µg/mL + 3.125 × 10^4^ particle/mL), and hypo-exo 10 (LPS 1 µg/mL + 1 × 10^6^ particle/mL). L2 cells were cultured in the DMEM high glucose medium. After the cells were 80%–90% confluent, the growth medium was replaced with DMEM low glucose medium containing LPS 1 µg/mL (Sigma-Aldrich, L2880, Germany) for inflammation induction and then incubated at 37°C with 5% CO_2_ for 24 hours. After 24 hours, nor-exo and hypo-exo were added and incubated at 37°C with 5% CO_2_ for 24 hours. L2 cells from treatment were used for TLR4 and NF-κB gene expression, and medium were used to measure IL-1β, TNF-α, and IL-6 levels using an ELISA assay.

###  Quantitative real-time PCR

 L2 cells were extracted using the RNA isolation kit (Zymo, R2073) according to the manufacturer’s protocol. RNA was extracted and reverse transcripted (50 μg RNA) to cDNA. Reverse transcripted to complementary DNA using an iScript Reverse Transcription Supermix for RT-PCR (Bio-Rad, 170-8841) according to the manufacturer’s protocol. The expression levels of TLR4 and NF-κβ were determined using the qRT-PCR SensiFAST^TM^ SYBR® No-ROX Kit (Bioline, BIO-98005, USA). Relative expression levels of TLR4 and NF-κβ were calculated using the 2−^ΔΔ^Ct method. Table 1 shows the primer sequences (Macrogen).

**Table 1:** Primer sequence

**Gene**	**Primer sequence (5′–3′)**	**Product size (bp)**	**Annealing (°C)**	**Reference**
TLR4	Forward: AGTGAGAATGCTAAGGTTGG Reverse: ATTAGGAAGTACCTCTATGCAG	289	54	NM_019178.2
NF-κβ-p65	Forward: GGACTATGACTTGAATGCGG Reverse: ACACCTCAATGTCTTCTTTCTG	230	54	NM_199267.2
GAPDH	Forward: TCAAGATGGTGAAGCAG Reverse: ATGTAGGCCATGAGGTCCAC	217	54	NM_001289726.1

###  TNF-α, IL-1β, and IL-6 level measurements

 Proinflammatory level measurement in L2 cells medium after treatment. TNF-α (Elabscience, E-EL-R2856, USA), IL-6 (Elabscience, E-EL-R0015, USA), and IL-1β (Elabscience, E-EL-R0012, USA) were detected by enzyme-linked immunosorbent assay (ELISA) using the microplate reader and absorbance was measured at 450 nm.

###  Proteomic analysis of hWJ -MSC-Exo

 Analysis of the amount of protein from exosomes derived from hWJ-MSCs treated with normoxia and hypoxia using liquid chromatography-mass spectrometry (LC-MS/MS) (Shimadzu, Japan). Nor-exo and hypo-exo were dissolved in mobile phase A asolvent (0.1% (v/v) formic acid solution) and delivered at 300 nL/min from a C18 trap column to an analytical column using a NanoElute ultra-high-performance liquid phase system. Mobile phase B was an acetonitrile solution containing 0.1% formic acid. Electrospray voltage was 2.0 kV. Orbitrap discovered intact peptides with 70 000 resolution from 350 to 1800 m/z.^16^

###  Bioinformatics analysis

 ProteoWizard software translated LC-MS/MS mzML data to txt. The estimated area proportionate Venn module diagram compared development times and discovered nor-exo and hypo-exo proteins. FunRich and Vesiclepedia were used to analyze exosome protein datasets for functional enrichment.^17^

###  Statistical analysis

 SPSS (IBM SPSS Statistics for Windows 22, IBM Corp., New York) was used for statistical analysis. The data collected were analyzed using ANOVA.

## Results and Discussion

###  Culture and identification of hWJ -MSCs

**Figure 1:**
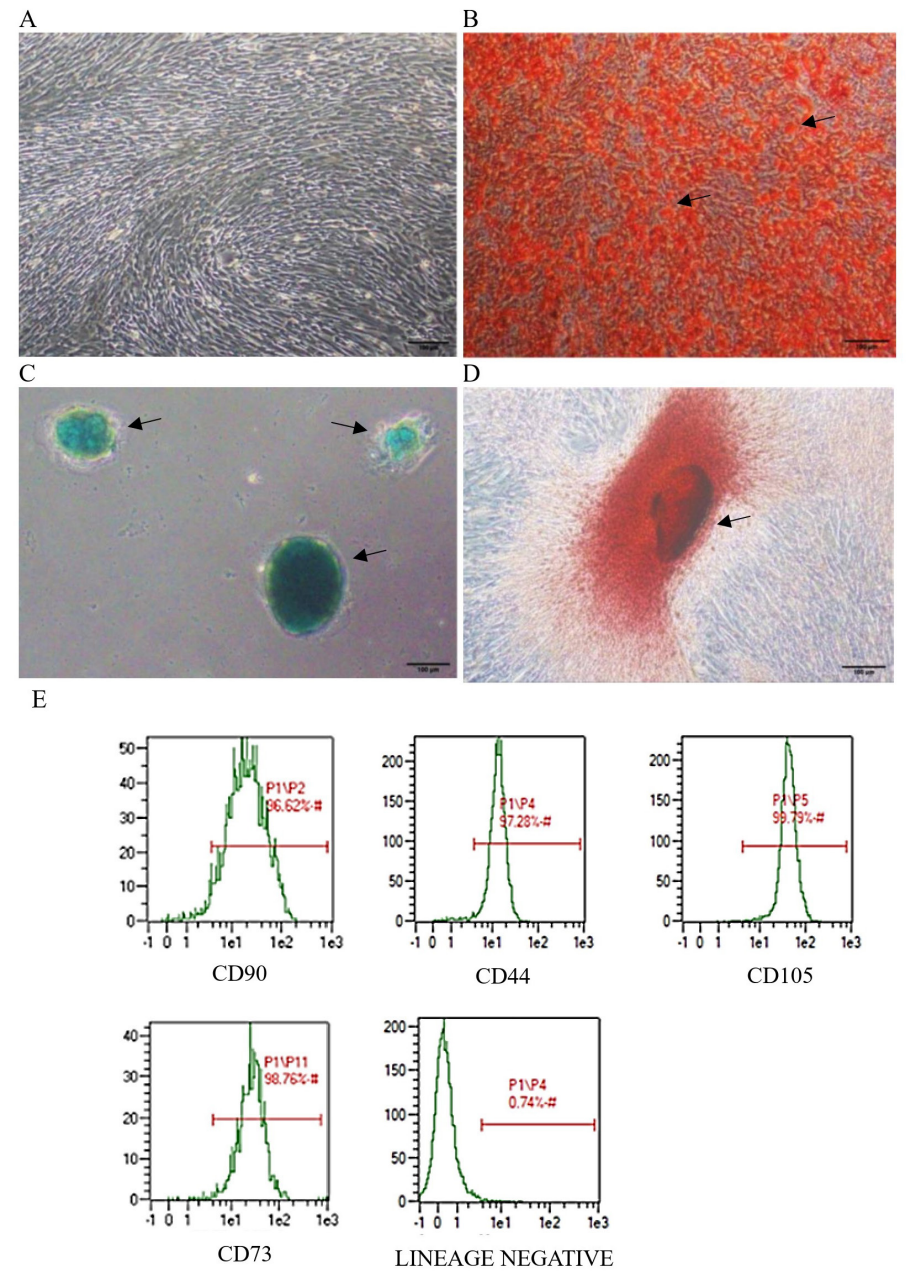
Trilineage differentiation and surface marker identification of hWJ-MSCs. (A) Primary hWJ-MSC morphology after 12 days of culture from WJ explant. Multipotency assay staining: (B) Adipogenic differentiation stained with oil red o; (C) Chondrogenic differentiation stained with alcian blue; (D) Osteogenic differentiation stained with alizarin red; (E) Analysis of positive MSC specific surface marker. Scale bars = 100 μm

 hWJ-MSCs showed a typical fibroblast-like morphology and adherent characteristics under an optical microscope (Figure 1). Lipid droplets, accumulation of GAG, and calcium deposits formed after staining the cells, indicating that hWJ-MSCs could differentiate into adipocytes, chondrocytes, and osteocytes. Flow cytometry showed that isolated hWJ-MSCs were positive for MSC markers: CD90, 96.62%; CD44, 97.28%; CD105, 99.79%; CD73, 98.76%; and Lin (−) negative marker, 0.74%. hWJ-MSCs at passage 4 (P4) were confirmed for use in obtaining exosomes. On the basis of these results, it was certain that these cells were MSCs.

###  Isolation and characterization of exosomes

**Figure 2:**
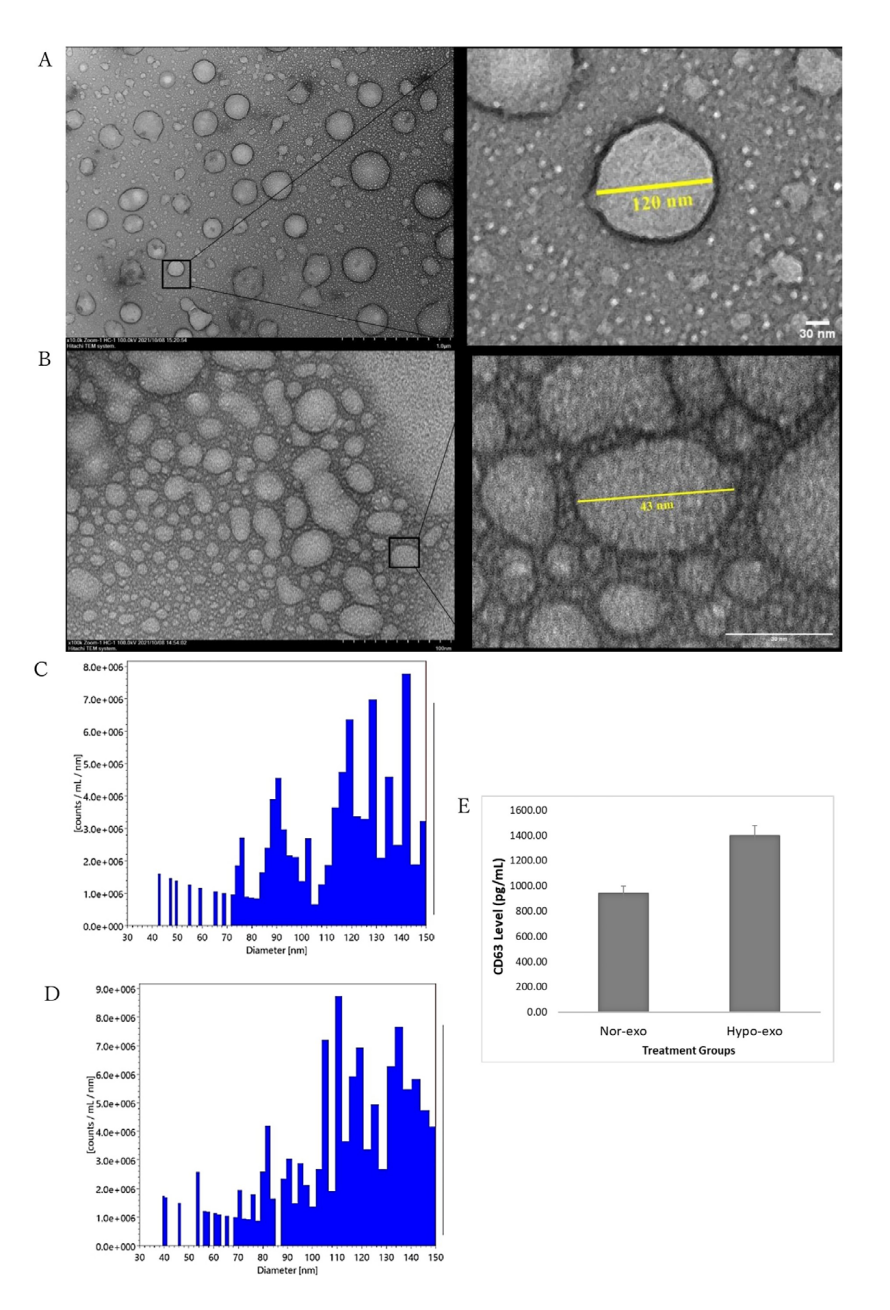
Characterization of exosomes isolated from hWJ-MSCs. (A) Transmission electron microscopy (TEM) of nor-exo. (B) Transmission electron microscopy (TEM) of hypo-exo. (C) Nanoparticle Tracking Analysis (NTA) of nor-exo. (D) Nanoparticle Tracking Analysis (NTA) of hypo-exo. (E) CD63 marker analysis of nor-exo and hypo-exo. Scale bars = 30 μm

 The morphologies of nor-exo and hypo-exo were successfully observed using transmission electron microscopy with negative staining (Figure 2A and Figure 2B). Furthermore, nanoparticle tracking analysis measurement was used to further assess particle size distribution and the number of nor-exo and hypo-exo particles The number of particles observed in the NTA was 1.3 × 10^8^ particle/mL with an average exosome size of 113 nm for nor-exo (Figure 2C) and 2.1 × 10^8^ particle/mL with an average exosome size of 114 for hypo-exo (Figure 2D). In this study, it was shown that hypo-exo yields were higher than nor-exo yields.

 Next is the characterization of the membrane proteins found in exosomes. Tetraspanins CD9, CD63, CD37, CD81, and CD82 are specially enriched in the membrane of exosomes, and they are often used as exosome biomarkers. Several studies describe CD63 and CD81 as the most frequently identified proteins in exosomes, and CD63 and CD81 are considered classical markers of exosomes.^18^ Exosome marker CD63 was used in this study. Figure 2E shows that nor-exo has a lower intensity (942.81 pg/mL) than that of hypo-exo (1398.05 pg/mL). The increase in CD63 in hypo-exo may be related to a higher number of particles based on the results of the nanoparticle tracking analysis compared with nor-exo. Proteins produced in hypo-exo were in greater numbers than those under normoxic conditions, leading to better biological pathways.^19^ Collectively, these data demonstrated that the characteristics of the exosomes extracted and purified from hWJ-MSC culture medium met the typical criteria for extracellular vesicles.

###  Exosome uptake by L2 cells

**Figure 3:**
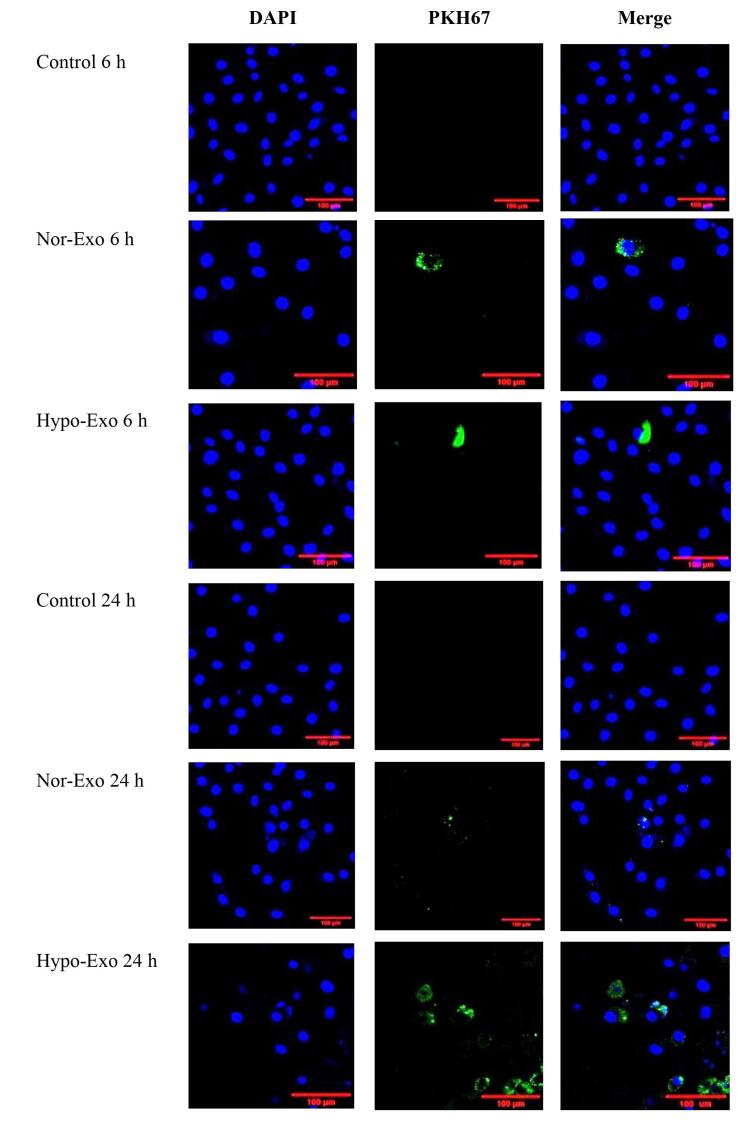
Nor-exo and hypo-exo on L2 cells. L2 cells were cultured for 6 h and 24 h and processed for exosome staining PKH67 (green) by confocal microscopy. DAPI (blue) was used to stain nuclei. Representative images are shown at 200 × . Scale bars = 100 μm

 To explore the reason that exosomes can have an anti-inflammatory role, laser scanning confocal microscopy was used to determine nor-exo and hypo-exo and distribution in L2 cells for cellular uptake. Nor-exo and hypo-exo from hWJ-MSCs successfully fused into target cells of interest, which was a prerequisite for performing their functions. Figure 3 shows that both nor-exo and hypo-exo can be internalized by L2 cells. At 6 hours, the exosomes only clumped together in a few cells. Therefore, it is possible to assume that the exosomes have entered at 6 hours despite the intensity not being as evenly distributed as that at 24 hours. These findings demonstrate the ability to transfer exosomes and their cargo between cells. This study shows that at 24- hour incubation, nor-exo and hypo-exo were internalized in L2 cells.

###  Relative gene expression of TLR4 and NF-κβ and inflammation marker levels in LPS-induced cell injury

**Figure 4:**
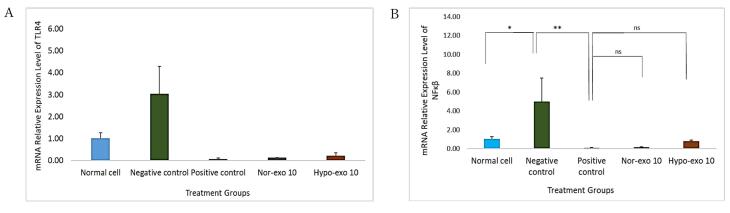
TLR4 and NF-κβ relative gene expression in L2 cells. (A) TLR4 relative gene expression, normal cells (untreated cells) & negative control (LPS 1 µg/mL) *(*P <*0.05), negative control (LPS 1 µg/mL) & positive control (LPS + Dex 40 ng/mL) **(*P <*0.05), positive control (LPS + Dex 40 ng/mL) & nor-exo 10 (LPS 1 µg/mL + 1 x 106 particles/mL) ns (*P* > 0.05), positive control (LPS + Dex 40 ng/mL) & hypo-exo 10 (LPS 1 µg/mL + 1 x 106 particles/ mL) ns (*P* > 0.05). (B) NF-κβ relative gene expression, normal cells (untreated cells) & negative control (LPS 1 µg/mL) *(*P <*0.05), negative control (LPS 1 µg/mL) & positive control (LPS + Dex 40 ng/mL) **(*P <*0.05), positive control (LPS + Dex 40 ng/mL) & nor-exo 10 (LPS 1 µg/mL + 1 x 106 particles/mL) ns (*P* > 0.05), positive control (LPS + Dex 40 ng/mL) & hypo-exo 10 (LPS 1 µg/mL + 1 x 106 particles/ mL) ns (*P* > 0.05). Data are presented as mean ± standard deviation. ns: no significance

 On the basis of the nor-exo and hypo-exo uptake on the L2 cell line, it is known that exosomes can penetrate cells and exert effects on cells. The mRNA relative expression of TLR4 was significantly higher in negative control compared than in normal cells (*P* < 0.05), after treatment with nor-exo 10 and hypo-exo 10 (1 × 10^6^ particles/mL), TLR4 gene expression significantly decreases and no significant with positive control (*P* < 0.05) (Figure 4A). As previously known, TLR4 activates NF-κβ signaling, so it is in line with the results of the TLR4 gene expression that negative control increases the expression of the NF-κβ gene compared to normal cells (*P* < 0.05). Apart from that, the administration of nor-exo 10 and hypo-exo 10 (1 × 10^6^ particles/mL) (*P* < 0.05) also reduces NF-κβ gene expression (Figure 4B).

 A cascade of LPS receptors and secondary proteins, including the LPS binding protein, CD14, and TLR4–MD-2 complex, can detect the typical structural pattern of LPS found in a wide variety of bacterial species.^20^ Alveolar, bronchial, and vascular endothelial cells express TLR4 in the lung.^21^ This study shows that L2 cells induced with LPS have a higher TLR4 relative gene expression compared with normal cells. This result in line with Sender and Stamme^22^ revealed that LPS modulated TLR4 gene and protein expression in type I and type II alveolar epithelial cells (AECI and AECII).

**Figure 5:**
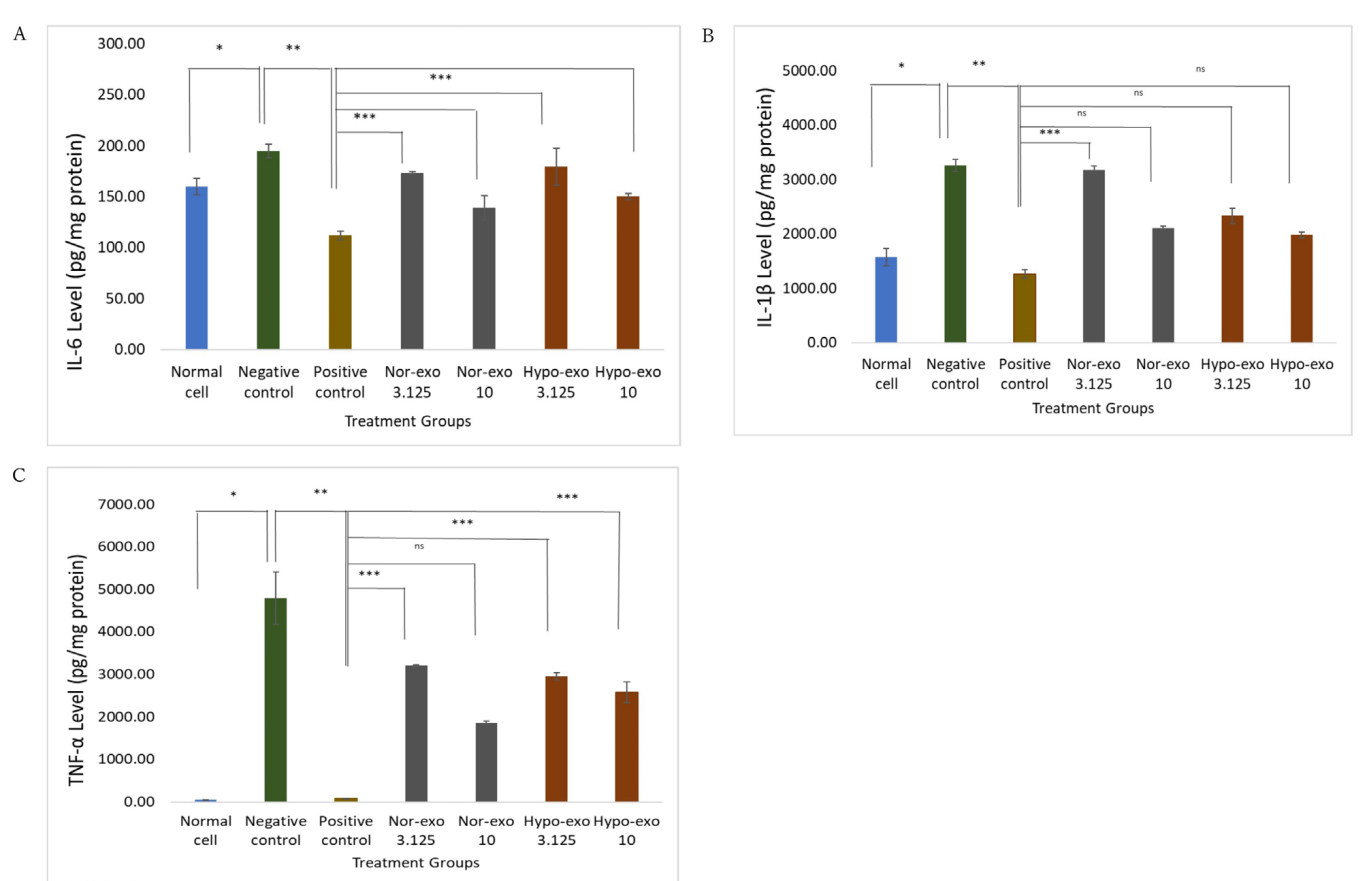
Effect nor-exo and hypo-exo toward pro-inflammatory cytokine in LPS-induced L2 cells. (A) IL-6 Level, normal cells (untreated cells) & negative control (LPS 1 µg/mL) *(*P <*0.05), negative control (LPS 1 µg/mL) & positive control (LPS + Dex 40 ng/mL) **(*P* < 0.05), positive control (LPS 1 µg/mL + Dex 40 ng/mL) & nor-exo 3.125 (LPS 1 µg/mL + 3.125 x 104 particles/mL) *** (*P <*0.05), positive control (LPS + Dex 40 ng/mL) & nor-exo 10 (LPS 1 µg/mL + 1 x 106 particles/mL) ns (*P* > 0.05), positive control (LPS + Dex 40 ng/mL) & hypo-exo 3.125 (LPS 1 µg/mL + 3.125 x 106 particles/ mL) *** (*P <*0.05), positive control (LPS + Dex 40 ng/mL) & hypo-exo 10 (LPS 1 µg/mL + 1 x 106 particles/ mL) ns (*P* > 0.05). (B) IL-1β Level, normal cells (untreated cells) & negative control (LPS 1 µg/mL) *(*P <*0.05), negative control (LPS 1 µg/mL) & positive control (LPS 1 µg/mL + Dex 40 ng/mL) **(*P <*0.05), positive control (LPS 1 µg/mL + Dex 40 ng/mL) & nor-exo 3.125 (LPS 1 µg/mL + 3.125 x 104 particles/mL) *** (*P <*0.05), positive control (LPS 1 µg/mL + Dex 40 ng/mL) & nor-exo 10 (LPS 1 µg/mL + 1 x 106 particles/mL) ns (*P* > 0.05), positive control (LPS 1 µg/mL + Dex 40 ng/mL) & hypo-exo 3.125 (LPS 1 µg/mL + 3.125 x 104 particles/ mL) ns (*P* > 0.05), positive control (LPS 1 µg/mL + Dex 40 ng/mL) & hypo-exo 10 (LPS 1 µg/mL + 1 x 106 particles/ mL) ns (*P* > 0.05). (C) TNF-α Level, normal cells (untreated cells) & negative control (LPS 1 µg/mL) *(*P <*0.05), negative control (LPS 1 µg/mL) & positive control (LPS 1 µg/mL + Dex 40 ng/mL) **(*P <*0.05), positive control (LPS 1 µg/mL + Dex 40 ng/mL) & nor-hypo 3.125 (LPS 1 µg/mL + 3.125 x 104 particles/mL) *** (*P <*0.05), positive control (LPS 1 µg/mL + Dex 40 ng/mL) & nor-hypo 10 (LPS 1 µg/mL + 1 x 106 particles/mL) ns (*P* > 0.05), positive control (LPS 1 µg/mL + Dex 40 ng/mL) & hypo-exo 3.125 (LPS 1 µg/mL + 3.125 x 104 particles/ mL) *** (*P <*0.05), positive control (LPS + Dex 40 ng/mL) & hypo-exo 10 (LPS 1 µg/mL + 1 x 106 particles/ mL) *** (*P <*0.05). Data are presented as mean ± standard deviation. ns: no significance

 In addition, induction of lipopolysaccharide (LPS) in L2 cells has been shown to increase IL-6, IL-1β, and TNF-α levels in normal cells. The results of this study showed that IL-6 concentrations increased after induction with LPS compared with normal cells. Dexamethasone administration could significantly reduce IL-6 concentrations to 111.89 ± 4.34 pg/mg protein compared to the negative control (*P* < 0.05, *P* = 0.002). On the basis of the four groups, administration of nor-exo 10 after LPS induction reduced IL-6 concentrations by 139.29 ± 12.11 pg/mg protein (*P* > 0.05, *P* = 0.254) and hypo-exo 10 by 150.14 ± 2.74 pg/mg protein (*P* > 0.05, *P* = 0.098), for those treatment no significant different to the positive control so it was equivalent to the positive control compared to nor-exo 3.125 and hypo-exo 3.125 (Figure 5A).

 Next, IL-1β concentration increased significantly after administration of LPS 1 µg/mL with a concentration of 3259.28 ± 112.68 pg/mg protein compared to normal cells of 1578.34 ± 156.90 pg/mg protein *P* < 0.05, *P* = 0.000). Administration of dexamethasone (positive control) to LPS-induced cells significantly reduced IL-1β concentration to 1264.28 ± 74.64 pg/mg protein (*P* < 0.05, *P* = 0.042). Treatment with hypo-exo 10 (*P*> 0.05, *P* = 0.166) and nor-exo 10 (*P* > 0.05, *P* = 0.687) had no significant difference with the positive control (Figure 5B).

 LPS induction in L2 cells showed a significant increase in the concentration of TNF-α to 4790.27 ± 613.67 pg/mg protein from normal cells, which was only 52.32 ± 0.12 pg/mg protein (*P* < 0.05, *P* = 0.050). Administration of dexamethasone can significantly reduce the concentration of TNF-α to 80.17 ± 5.30 pg/mg protein, approaching normal cells (*P* < 0.05, *P* = 0.050). On the basis of the four treatment groups, nor-exo 10 had a TNF-α concentration of 1865.19 ± 42.92 pg/mg protein (*P* > 0.05, *P* = 0.513), which was not significantly different to the positive control compared to nor-exo 31.25, hypo-exo 10, and hypo-exo 3.125 (Figure 5C). The best concentration for decreasing IL-6 and TNF-α was nor-exo 10 (1 × 10^6^ particles/mL), but hypo-exo 10 (1 × 10^6^ particles/mL) was the best concentration for decreasing IL-1β. However, in this study, all treatments for nor-exo and hypo-exo significantly reduced IL-1β, TNF-α, and IL-6 levels.

**Figure 6:**
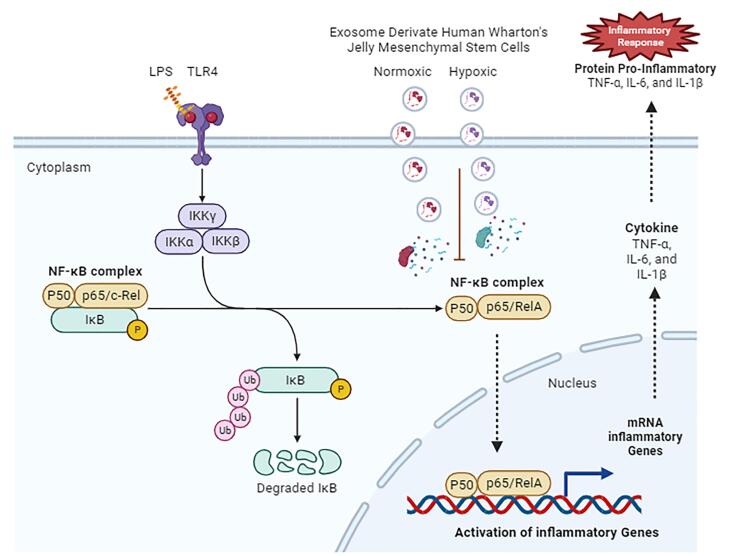
Hypothetical diagram of the mechanism in inhibition of pro-inflammatory cytokines by human Wharton’s Jelly mesenchymal stem cell-derived exosomes

Figure 6 illustrates how hWJ-MSC exosomes affect lipopolysaccharide-induced lung injury. TLR4 activates NF-κβ and induces proinflammatory cytokines.^21^ MSCs transmit functional cargos to recipient cells via exosomes to treat diseases. Exosome components, such as proteins, peptides, lipids, cytokines, mRNAs, and miRNAs, are released into the cytoplasm by membrane fusion or endocytosis and change gene expression and signal transduction pathways in recipient cells.^23^ Exosomes from hWJ-MSCs inhibit NF-κB dissociation from IκB, resulting in NF-κB nuclear translocation inhibition and decreased expression of its target genes encoding of inflammatory cytokines.

 TNF-α, IL-1β, and IL-6 attract and activate inflammatory cells in acute lung injury (ALI). TNF-α activates neutrophils, and IL-6 is a biomarker of lung neutrophil accumulation and tissue injury.^24,25^ Exosomes derived from MSCs prevent inflammatory cells from entering the injury site, reduce lung damage, and decrease inflammatory cytokines, including TNF-α and IL-6.^26^ Intratracheal MSC-MVs reduced lung inflammation in a mouse model of LPS-induced ALI.^27^

 According to this study, nor-exo and hypo-exo have the capability to suppress TLR4 and NF-κB gene expression and to reduce proinflammatory cytokines. Thus, hypoxia induction in hWJ-MSCs to obtain exosomes only increases the yield of exosomes and does not affect their biological role.

###  Bioinformatics analyses of exosomes

**Figure 7:**
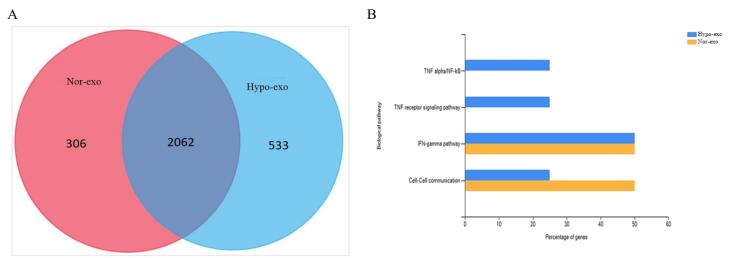
Proteomic analysis of nor-exo and hypo-exo. (A) Liquid chromatography–tandem mass spectrometry (LC-MS/MS) analysis of nor-exo and hypo-exo proteins. FunRich software and the Vesiclepedia database analyzed LC-MS/MS data. (B) Proteins are abundant and play a role in nor-exo (yellow) and hypo-exo (blue)

 Exosome analysis using LC-MS/MS analysis revealed the identification of 2368 proteins in nor-exo and 2595 proteins in hypo-exo (Figure 7A). The protein-protein interaction (PPI) network helps us understand the function of the proteome and the protein load of exosomes found in nor-exo and hypo-exo, as in this study four PPI networks were found that are related to anti-inflammatory (Figure 7B). This study discovered interesting pathways associated with inflammation by focusing on the PPI networks derived, such as cell–cell communication, TNF receptor signaling pathway, TNF-α/NFκβ, IL-5 mediated signaling events, and IFN gamma pathway.

 On the basis of the proteomic result, nor-exo and hypo-exo have the potential as an anti-inflammatory therapy. In the exosome load, various proteins were identified. Furthermore, decreased NF-κB activity measured by p65 levels decreases IL-1β, TNF-α, and IL-6 levels and lung tissue damage.^28^ The protein present in nor-exo and hypo-exo also has a biological pathway. Under hypoxic conditions, MSCs enriched their cargo with anti-inflammatory cytokines and angiogenesis-related proteins as mentioned in de Jong et al.^29^

 Overall, the results of this study can be summarized in several points. hWJ-MSCs-Exo can be characterized and can produce higher yields under hypoxic conditions, which can be further increased in terms of isolation production from exosomes, which leads to a better profile of exosome function as an anti-inflammatory agent. Although the biological role of hypo-exo is as good as that of nor-exo as shown in the results of this study, conditioning with the hypoxic on hWJ-MSCs can be a method to increase the yield of exosomes. Further research is required to increase the production of exosomes and analyze several further inflammatory markers to achieve a more comprehensive understanding of the inflammatory pathways involved in lung injury.

## Conclusion

 According to the findings of this study, nor-exo and hypo-exo derived from hWJ-MSCs have anti-inflammatory activities. Hypo-exo has the advantage of increasing exosome yield and has a higher protein profile. However, hypo-exo does not improve the biological function compared to nor-exo. A decrease in IL-6, IL-1β, and TNF-α levels, as well as a decrease in TLR4 and NFκβ gene expression, indicated an anti-inflammatory effect. Proteomic analysis results supported the above data and gave a new insight for further study. This study suggests that exosomes derived from hWJ-MSCs under a hypoxic condition might be developed further as future anti-inflammatory agents in cell-free-based therapy.

## Acknowledgments

 The author is very grateful for the research funding support provided by Riset ITB 2021 with research grant number 148/IT1.B07.1/TA.00/2021. This research was supported by AMU-BBRC, Bandung, Indonesia. The authors would also like to thank Dr. Wahyu Widowati, M.Si., Seila Arumwardana, Cahyaning Riski Wijayanti, Meganita Marthania, and Agung Novianto for their valuable assistance.

## Competing Interests

 The authors have no conflict of interest.

## Ethical Approval

 All experimental procedures were approved by the Medical Research Ethics Committee of Universitas Padjajaran, Indonesia (approval letter no. 477/UN6.KEP/EC/2021; date of approval: June 09, 2021).
